# Investigations into the occurrence of polycyclic aromatic hydrocarbons in surface waters of the Narok and Bomet counties of Kenya

**DOI:** 10.1098/rsos.240019

**Published:** 2024-10-09

**Authors:** Bakari Chaka, Aloys M. Osano, Omwoyo Wesley Nyaigoti, Patricia B. C. Forbes

**Affiliations:** ^1^ Department of Mathematics and Physical Sciences, Maasai Mara University, P.O. Box 861-20500, Narok, Kenya; ^2^ Department of Chemistry, Faculty of Agriculture and Natural Sciences, University of Pretoria, Pretoria 0001, South Africa

**Keywords:** PAHs, eco-toxicity, pollution, hazardous

## Abstract

Polycyclic aromatic hydrocarbons (PAHs) are a group of emerging chemical pollutants that pose severe health challenges and toxicity to people and aquatic organisms exposed to these pollutants. This study sought to assess the types and levels of PAHs and their eco-toxicity indices in surface waters of Narok and Bomet counties of Kenya, which have witnessed an increase in charcoal-burning activities and vehicular emissions near water bodies. Sampling was done in eight regions of the two counties based on their proximity to PAH sources. Extraction of the water samples was done via a solid-phase mmethod. Seven US Environmental Protection Agency (US EPA) priority PAHs were detected. The concentrations of these PAHs varied from below the limits of detection up to 31.42 µg l^−1^ for dibenzo[*a,h*]anthracene. The majority of the PAHs from Narok County were pyrogenic, while those from Bomet were petrogenic based on PAH diagnostic ratios. The surface waters were significantly polluted with dibenzo[*a,h*]anthracene, with risk quotients above 1.0 in the surface waters, and were found to be hazardous, with hazard quotients above 10.0, thus indicating potential environmental risks. The findings indicate the need for stringent measures to be put in place to mitigate the risks posed by these PAHs.

## 1. Introduction

Polycyclic aromatic hydrocarbons (PAHs) are a group of xenobiotic compounds of interest [1–3]. PAHs are found almost everywhere—in the atmosphere, water and soil as well as in food [4], all of which people interact with in their day-to-day lives. They are also challenging to control owing to the multiple sources releasing them into the environment [5] both naturally and via anthropogenic activities. PAHs can be released naturally through, for example, volcanic eruptions [6]. PAHs are also released via human activities such as oil seepage and exploration activities, vehicular emissions, domestic heating (incineration and burning of wastes), cooking in poorly ventilated kitchens, and charcoal-burning [7]. Narok and Bomet counties are found in the southern part of the Mau Forest—the region’s largest water catchment source [8]. Increased anthropogenic activities in and around the forest have led to gradual reduction of the forest to almost extinction [9]. One of the persistent human activities in this area is charcoal-burning [9]. This activity is done for commercial purposes (source of livelihood to charcoal burners) as well as for domestic fuel. At least 30% of residents in these two counties depend on charcoal for cooking (60 and 35% of urban and rural dwellers, respectively) [10]. This activity is a potential contributors to PAHs in the region. The residents of these counties as well as their livestock consume water directly, with minimal purification, thus being strongly susceptible to PAHs toxicity. It is for this reason that there is a need to monitor the levels and types of PAHs in the counties for effective mitigation measures to be planned.

A wide range of PAHs have been detected in water and sediments [11,12]. PAHs are relatively soluble in water, with their lipophilicity decreasing with molecular weight [13]. These compounds are made up of carbon and hydrogen in two or more fused aromatic rings. The molecular weight of PAHs is quite significant since it not only affects their lipophilicity but also their atmospheric mobility and volatility [1]. PAHs in water associate freely with dissolved organic matter via binding and adsorption [14–18], which may lead to aquatic toxicity. Bio-availability of PAHs in water and consequent exposure of animals though food and water has thus become a global ecological challenge [19,20].

PAHs pose serious health challenges, with several of them being classified as carcinogenic, mutagenic and teratogenic [21].

At least 16 of these compounds have been ranked as being priority ecological pollutants by the United States Environmental Protection Agency (US EPA). The compounds include anthracene, benzo[*a*]pyrene, benzo[*g,h,i*]perylene, dibenz[*a,h*]anthracene, fluoranthene, naphthalene and phenanthrene. Out of these, benzo[*a*]pyrene and dibenzo[*a,h*]anthracene are carcinogenic as per the International Agency for Research on Cancer [22,23].

Owing to the risk these pollutants may pose, and the possible routes into water bodies, especially from charcoal-burning in the study region, this study sought to determine the concentrations of the US EPA pollutants in surface waters in the two counties and thereby better inform best management practices. This study sought to provide a spatial distribution of PAHs in surface waters in Narok and Bomet counties, Kenya. This is due to the presence of charcoal-burning activities in the Mau Forest region, which is the water catchment for these counties [8]. The residents of the counties and their livestock heavily rely on these surface waters for consumption [24]; thus there is an ecological risk should the water be polluted with PAHs.

## 2. Material and methods

**Figure 1.**
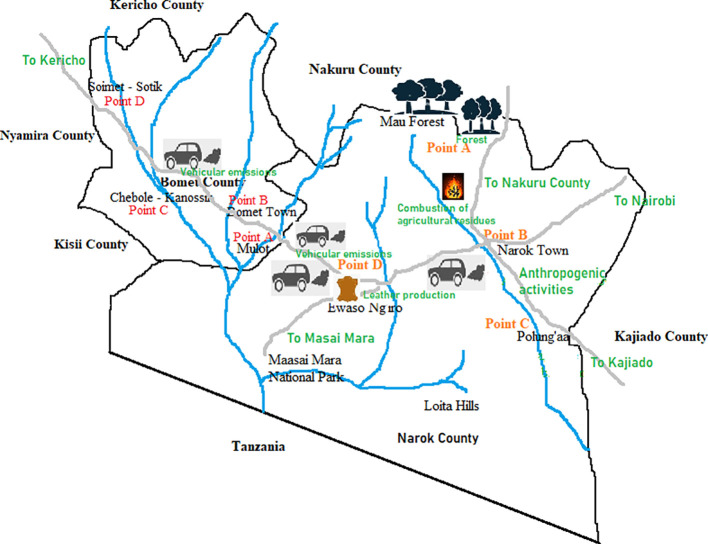
The sampling sites in Narok County and Bomet County.

A randomized one-factorial research design focusing on proximity of point-source pollutants to the surface waters was used for the study. Sampling was done in Narok and Bomet counties between 15 and 22 October 2022 based on the geographical proximity to anthropogenic activities that lead to PAH emissions. Four sampling sites located in different parts of Narok and Bomet counties were identified, and samples were collected from each of the counties (figure 1). At least four sampling points were selected for each of the two counties. At each sampling point, triplicate sampling was done separated by a distance of 1 m and with a river depth of 1.0–1.5 m. The samples were then homogenized to form a composite sample for the sampling point. Grab sampling was used to collect 500 ml aliquots of the surface water samples using light-proof 500 ml polyethylene terephthalate (PET) bottles with high-density polyethylene (HDPE) caps. Physico-chemical properties of the samples were determined *in situ* using a portable pH meter (Hanna G-114, Shimadzu) calibrated with pH 4.0, 7.0 and 10.0 commercial buffer solutions (Testo, Kenya) and an electrical conductivity (EC), dissolved oxygen (DO) and turbidity meter (Hach HQ440D, USA). The procedure involved measuring a 10 ml aliquot of distilled water as a blank before analysing the water samples in triplicates and the mean value taken. Gradual agitation of the samples using the pH and conductivity, oxygen and turbidity meters, respectively, was done before the readings were taken, to ensure maximum capture of the ions present. A drop of 1% nitric acid was then added to the samples and they were transferred in a cooler box to the Kenya Revenue Authority (KRA) laboratories for analysis.

### 2.1. Extraction

Prior to extraction, the samples were filtered using 0.25 µm hydrophilic polyethersulfone (PES) membrane syringe filters (Millex-GP). The samples were then concentrated using a solid phase extraction (SPE) cartridge (HLB Oasis) and an SPE manifold. This involved prior conditioning of the SPE cartridges with 10.0 ml methanol followed by 5.0 ml deionized water. Thereafter, 100.0 ml of the samples was loaded and washed with 5.0 ml de-ionized water to remove contaminants. The samples were then concentrated in the cartridges to 10.0 ml before extraction [25]. PAH extraction procedures were then conducted as described by Kanchanamayoon & Tatrahun [26]. The SPE columns were conditioned with 5 ml of methanol followed by 5 ml of de-ionized water. A 10 ml aliquot of de-ionized water was spiked at 0.1 ppb with a US EPA PAH multi-residue surrogate standard (containing the 16 US EPA priority PAHs) (99.999% pure, Dr Ehrenstorfer’s, Ausburg, Germany) and passed through the manifold at a flow rate of 1 ml min^−1^. The SPE column was then dried at 25°C before elution with isooctane : methylene chloride (1 : 4). The extracts were concentrated to a final volume of 0.2 ml under a gentle stream of nitrogen as described by Fernández-Espinosa [27].

### 2.2. Analysis

Analysis was done according to the method by Elaridi *et al.* [28]. The samples were analysed for PAHs using a gas chromatograph, GC (Perkin Elmer, Clarus 680, UK), coupled to mass spectrometer, MS, (Perkin Elmer, Clarus 8T). The GC had a (5%-phenyl)-methylpolysiloxane (low polarity) capillary column (30 m × 0.25 mm internal diameter × 0.25 μm film thickness) from Agilent Technologies. The carrier gas was helium (99.999% purity) at a flow rate of 1 ml min^−1^. Aliquots of 2.0 µl were injected in a splitless mode starting at 200 psi for 0.3 min. The temperature of the oven was set to 45°C for 0.8 min then gradually increased to 300°C then held for 5 min in isothermal mode at a ramp rate of 10°C min^−1^. The column flow rate was 1.08 ml min^−1^, linear velocity 37.8 cm s^−1^ at a pressure of 11.3 psi.

The MS source temperature was maintained at 280°C with a solvent vent time of 4.5 min. Electron ionization (EI) was used. The quadrupole temperature was maintained at 180°C with a detector voltage set at 1500 V. The transfer line and ion source temperatures were 280 and 230°C, respectively. For calibration, a stock solution of 1 mg l^−1^ of the standard mix was prepared in toluene. A mixed intermediate standard was then prepared at 500 ng ml^−1^ by dilution of the stock solution using methanol. A linear relationship was found in the range of 10–500 μg l^−1^, as illustrated in Appendix 1 (deposited in Dryad [29]). The limit of detection (LOD) and limit of quantification (LOQ) values of the standards were obtained from calculations based on the signal-to-noise ratio of 3 and 10, respectively.

### 2.3. Diagnostic ratios for polycyclic aromatic hydrocarbon sources

PAH diagnostic ratios are a tool to monitor and relate PAHs in relation to their molecular weights and probable emission sources [30]. The parent PAHs and proportion of alkyl-substitution to non-substituted molecules are considered using some of the notable PAHs such as anthracene, phenanthrene, fluoranthene and pyrene. The sources of PAHs were determined based on the following diagnostic ratios in equations (2.1)–(2.3) according to Janoszka *et al.* [31]:


(2.1) $$\frac{Ant}{Ant+Phen}=y_{1},$$

(2.2) $$\frac{Flu}{Flu+Py}=y_{2},$$

(2.3) $$\frac{LMW\text{-}PAH}{HMW\text{-}PAH}=y_{3},$$

where Ant is anthracene, Phen is phenanthrene, Flu is fluoranthrene, Py is pyrene, LMW-PAH is low-molecular-weight PAH, HMW-PAH is high-molecular-weight PAH and *y* is the diagnostic ratio. When *y*_1_ < 0.1, *y*_2_ < 0.4 and *y*_3_ > 1, the source of the PAHs is pyrogenic (arising from combustion of biomass such as charcoal-burning activities). When *y*_1_ > 0.1, *y*_2_ > 0.4 and *y*_3_ < 1, the source of the PAHs is petrogenic (arising from petroleum-based compounds such as coal and tar).

### 2.4. Ecological risk assessment of polycyclic aromatic hydrocarbons in water

The ecological risk assessment of the samples in the surface waters was conducted according to the method by Liang *et al.* [32]. Risk quotients (RQ) were used as discussed by Cao *et al.* [33]. RQ was calculated from the actual observed concentrations compared with reference concentration values (corresponding quality value) as indicated in equation (2.4):


(2.4) $$RQ=\frac{Cfield}{CQualityValue},$$

where *C*_field_ is the observed sample concentration and *C*_quality value_ is the permissible concentration of the PAHs obtained from standard measurements based on probabilistic models, usually at 95% percentile. An RQ of 0.01 ≤ RQ ≤ 0.1 indicates low levels of pollution, 0.1 ≤ RQ ≤ 1 indicates moderate pollution and 1 ≤ RQ indicates high pollution levels [32].

The tiered method was used to evaluate the ecological risk in the rivers as per the method by Jin *et al.* [34] and toxicology data from Qin *et al.* [35]. The no-observed-effect concentration (NOEC) of the PAHs was obtained from [36]. To determine effect on aqueous life, the hazardous concentration affecting at least 5% of the aquatic species (HC_5_) was calculated. If more than one value was available for these species, geometric means were calculated and used to plot the log–linear relationship curve () as shown in equation (2.5):


(2.5) $$y=\frac{1}{1+\text{exp}\left(a-x\right)/b},$$

where *y* is the calculated RQ value, *x* is the field concentration value of the samples, *a* is the mid-level HC_5_ calculated using the Bayesian Matbugs Calculator (BMC) method as described by He *at al.* [37], while *b* is the predicted no-effect concentration (PNEC) based on the quantitative structure–activity relationships (Q-SAR) method as described by Wang *et al.* [38,39]. The PNEC was calculated by dividing the HC_5_ by the assessment factor (AF), as indicated in equation (2.6):


(2.6) $$PNEC=\frac{HC_{5}}{AF},$$

where AF was obtained from [40]. The hazard quotient (HQ) of the PAHs was then determined by equation (2.7):


(2.7) $$HQ=\frac{Concn\;PAHs}{PNEC}.$$

The preliminary risk assessment ranks of PAHs were classified as insignificant if HQ < 0.1, low risk if 0.1 ≤ HQ < 1, moderate risk if 1 ≤ HQ < 10 and high risk if HQ ≥ 10 as described by Liu *et al.* [41].

## 3. Data analysis

The means, s.d. and coefficient of variation data of the PAHs obtained were analysed using GraphPad Prism, v. 9.5.0, 2022 and Microsoft Excel, v. 2019. A 95% confidence level was used for significance studies (*p ≤* 0.05; *n* = 3).

## 4. Results

**Figure 2.**
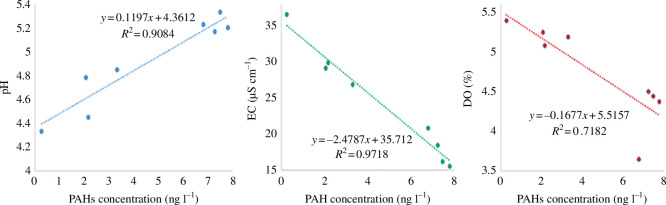
Correlation between polycyclic aromatic hydrocarbon (PAH) levels and selected physico-chemical properties of the surface waters of Narok and Bomet counties. EC, electrical conductivity; DO, dissolved oxygen.

**Figure 3.**
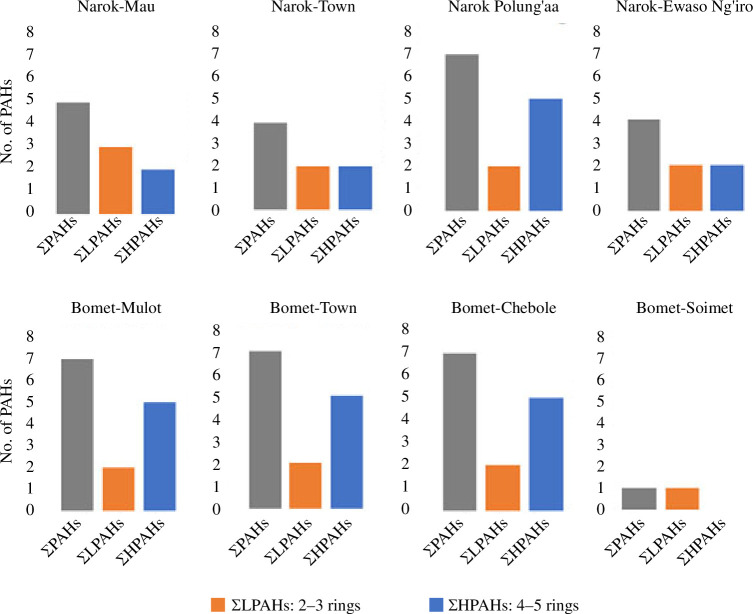
The distribution of low- and high-molecular-weight polycyclic aromatic hydrocarbons (PAHs) in Narok and Bomet counties, based on number of PAHs detected above the limit of detection (LOD).

**Figure 4.**
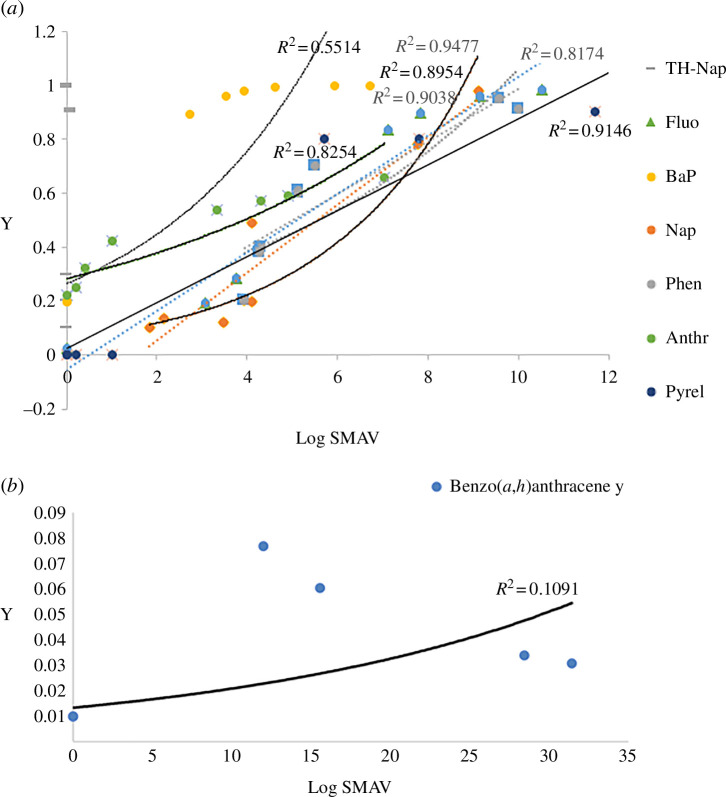
Species sensitivity distribution (SSD) plots for the detected PAHs (*a*) and for dibenzo[*a,h*]anthracene (*b*) in Narok and Bomet counties.

The findings of the study are highlighted in tables (1–3) and figures 1–4.

**Table 1.** Distribution of the polycyclic aromatic hydrocarbons (PAHs) detected within the study region. NA, Narok-Mau; NB, Narok-Town; NC, Narok Polung’aa; ND, Narok-Ewaso Ng’iro; BA, Bomet-Mulot; BB, Bomet-Town; BC, Bomet-Chebole; BD, Bomet-Soimet.

PAH	concentration of samples >LQ^a^ (µg l^−1^)														no. cases >LOD
	Narok							Bomet							
	NA	NB	NC	ND	mean	s.d.	CV (%)	BA	BB	BC	BD	mean	s.d.	CV (%)	
naphthalene	<LQ	< LQ	<LQ	<LQ	<LQ	<LQ	<LQ	<LQ	<LQ	<LQ	<LQ	<LQ	<LQ	<LQ	8
phenanthrene	<LQ	<LQ	<LQ	<LQ	<LQ	<LQ	<LQ	<LQ	<LQ	<LQ	<LQ	<LQ	<LQ	<LQ	7
fluoranthrene	<LQ	<LQ	<LQ	<LQ	<LQ	<LQ	<LQ	10.52	<LQ	<LQ	<LQ	10.52	<LQ	<LQ	7
anthracene	<LQ	<LQ	<LQ	<LQ	<LQ	<LQ	<LQ	<LQ	<LQ	<LQ	<LQ	<LQ	<LQ	<LQ	4
benzo[*a*]pyrene	<LQ	<LQ	<LQ	<LQ	<LQ	<LQ	<LQ	<LQ	<LQ	<LQ	<LQ	<LQ	<LQ	<LQ	7
benzo[*g*,*h*,*i*]perylene	<LQ	<LQ	15.85	<LQ	15.85	<LQ	<LQ	<LQ	<LQ	13.99	<LQ	13.99	<LQ	<LQ	4
dibenzo[*a*,*h*]anthracene	<LQ	<LQ	15.56	<LQ	15.56	<LQ	<LQ	12.01	31.42	29.42	<LQ	24.283	10.67	43.96	4

^a^ <LQ are values below the limit of quantification (LOQ) but above the limit of detection (LOD) values of the PAHs (see Appendix in [29]).

**Table 2.** Polycyclic aromatic hydrocarbon (PAH) diagnostic ratios and probable source at each sampling site in the study regions.

sampling point	PAH diagnostic source ratio			probable source
	$\frac{Ant}{Ant+Phen}$	$\frac{Flu}{Flu+Py}$	$\frac{LMW}{HMW}$	
Narok-Mau	0.00	1.00	1.00	pyrogenic
Narok-Town	0.00	1.00	1.00	pyrogenic
Narok-Polung’aa	5.27	1.00	2.50	petrogenic
Narok-Ewaso Ng’iro	0.00	1.00	1.00	pyrogenic
Bomet-Mulot	6.51	1.00	2.50	petrogenic
Bomet-Town	4.94	1.00	2.50	petrogenic
Bomet-Chebole	6.14	1.00	2.50	petrogenic
Bomet-Soimet	0.00	0.00	1.00	pyrogenic

Narok-Mau is ‘Narok Point A’, Narok-Town is ‘Narok Point B’, Narok-Polung’aa is ‘Narok Point C’, Narok-Ewaso Ng’iro is ‘Narok Point D’, Bomet-Mulot is ‘Bomet Point B’, Bomet-Town is ‘Bomet Point B’, Bomet-Chebole is ‘Bomet Point C’ and Bomet-Soimet is ‘Bomet Point D’ as illustrated in figure 1.

**Table 3.** The eco-toxicity values of the polycyclic aromatic hydrocarbons (PAHs) detected in Narok and Bomet surface waters. BMC, Bayesian Matbugs Calculator ecotoxicity values; Q-SAR, quantitative structure–activity relationship ecotoxicity values; PNEC, predicted no-effect concentration; HC_5_, the hazardous concentration affecting at least 5% of the aquatic species; RQ, risk quotient; HQ, hazard quotient.

PAH	BMC	Q-SAR	PNEC (µg l^−1^)	HC_5_ (µg l^−1^)	RQ		HQ	
					Narok	Bomet	Narok	Bomet
naphthalene	324.4	0.430	17.42	87.096	0.000	0.000	0.000	0.000
phenanthrene	106.2	7.460	1.300	13.00	0.000	0.000	0.000	0.000
fluoranthene	4.967	1.320	0.007	0.006	0.000	0.167	0.000	0.688
benzo[*a*]pyrene	1.090	0.770	0.0002	0.0002	0.131	0.123	20.82	19.53
anthracene	2.180	7.460	0.100	1.000	0.000	0.000	0.000	0.000
benzo[*g,h,i*]perylene	3.440	0.011	0.0008	0.0008	0.000	0.000	0.000	0.000
dibenzo[*a,h*]anthracene	69.00	0.011	0.0001	0.140	1.081	4.990	27.79	128.3

## 5. Discussion

The surface waters sampled in the two counties were found to be highly contaminated with at least eight types of low-molecular-weight (LMW) and high-molecular-weight (HMW) PAHs, ranging from <LOQ to 31.42 µg l^−1^. The HMW PAHs were attributed to petrogenic activities such as vehicular emissions and were more prevalent in Bomet County. Naphthalene was found to be the most prevalent PAH that was detected at all the sites and had a relatively mild toxicity. On the contrary, benzo[*a*]pyrene exhibited the highest eco-toxicity and was detected in both counties.

### 5.1. Polycyclic aromatic hydrocarbon occurrence and distribution

A total of eight PAHs were detected in the surface waters of both counties (table 1). The identified PAHs include naphthalene, phenanthrene, benzo[*a*]pyrene, fluoranthrene, anthracene, benzo[*g,h,i*]perylene and dibenzo[*a,h*]anthracene. Naphthalene was the most common PAH, being detected in all the regions studied. This is attributed to the numerous anthropogenic activities that lead to emission of PAHs—most being small-scale pyrogenic practices such as smoking cigarettes, and incomplete combustion of charcoal and firewood fuels as well as vehicular emissions [32]. These activities are uncontrolled and spread throughout the study region as illustrated in figure 1, thus explaining the presence of naphthalene in this area. Additionally, naphthalene is the smallest and most soluble PAH, and thus it is present in surface water as opposed to partitioning into sediments. Pyrogenic activities such as biomass combustion in the region were cited as the key source of the PAHs [42]. Naphthalene derivatives (1-methyl naphthalene, biphenyl, 2,3-dimethylnaphthalene and 1,4,5-trimethylnaphthalene) were detected in appreciable concentrations of 0.12 ± 0.03, 0.21 ± 0.04 and 0.060 ± 0.01 μg g^−1^ dry weight (dw) in river sediments in a study conducted by Opuru *et al.* [42] on the northern side of Mau Forest, in Elburgon River, Nakuru County. The region Bomet-Soimet, located in the tea plantation area in Sotik sub-county of Bomet, had the least number of PAHs detected i.e. only naphthalene. There were minimal charcoal burning activities upstream of the surface waters at this point, and the only PAH emission source here was anthropogenic activities such as use of wood fuel in the tea factories. There were also limited and scattered human settlements around the sampling point as the majority of the vast lands were occupied by tea plantations and other vegetation cover. Plants have been found to provide sinks for PAHs via conjugation and immobilization [43], thus potentially contributing to the reduced number of detected PAHs in Sotik.

Three-ringed PAHs, specifically phenanthrene and anthracene, were detected at all the sampled points except in Narok-Mau and Bomet-Soimet regions. Like naphthalene, these PAHs are also water-soluble and ubiquitous in nature [1]. Dibenzo[*a*,*h*]anthracene was recorded at the highest concentration of up to 28.42 and 31.32 ng l^−1^ in Bomet-Town and Bomet-Chebole, respectively. All the points at which this PAH was detected had a concentration well above 0.3 ng l^−1^—the maximum permissible concentration for dibenzo[*a*,*h*]anthracene as per the US Department of Health and Human Services (Agency for Toxic Substances and Disease Registry) standard (atsdr.dc.gov, 2013) [44] The surface waters were quite muddy, thus enabling detection of benzo[*a*]pyrene, which spends more time in sediments [45,46].

The high concentration of dibenzo[*a,h*]anthracene at these points was attributed to soot from vehicular emissions and tobacco smoke [47]. Benzo[*g,h,i*]perylene was also detected in the surface waters of Narok-Polungaa, Bomet-Mulot, Bomet-Town and Bomet C in concentrations of 15.89 ng l^−1^. Like dibenzo[*a,h*]anthracene, benzo[*a*]pyrene and benzo[*g,h,i*]perylene are five-ringed PAHs and thus have lower solubility in water and easily partition into sediments [1]. This explains why these PAHs were not detected at half of the points sampled. The points Narok-Polung’aa, Bomet-A, Bomet-Town and Bomet-Chebole registered the highest number of PAHs detected, i.e. seven types each. This is attributed to the multiple PAH sources at these points, with all points having charcoal-burning activities, dense human settlements and proximity to busy traffic producing vehicular emissions [48]. The points Bomet-Chebole and Narok-Polung’aa had a greater number of PAHs detected—possibly indicating more pollution in these regions. These findings were similar to those reported by Basweti *et al.* [49] in water (max.: 0.092 ± 0.003 ng l^−1^) arising from industrial activities in the River Nzoia, Kakamega County (Kenya). Shitandayi *et al.* [50] also observed 14 PAHs in the concentration range of 0.6–80 µg l^−1^ in waters of the Nzoia catchment region. These findings were also higher than those obtained by Ambade *et al.* [51] for river water and sediments from selected sites in the Subarnarekha River estuary, India.

### 5.2. Correlation between occurrence of polycyclic aromatic hydrocarbons and physico-chemical properties of the water

The pH and EC of the water samples were found to exhibit a linear relationship with the total PAH levels in the water at *r* ^2^ = 0.908 and 0.972, respectively. However, there was a weak linear correlation between DO and PAH concentration (*r* ^2^ = 0.718). A relationship between PAHs and the water pH level was found, with the concentration of PAHs increasing with pH level (figure 2). This is a crucial indictor for determining the nature of chemicals or filters to be used in elimination of PAHs from water. Similar findings were obtained by Batchamen Mougnol *et al.* [52], who also observed a similar trend between PAHs released in wastewaters and the pH levels of the water [53]. In the current study, naphthalene and phenanthrene were the most detected PAHs in the acidic samples—owing to their LMW and thus solubility [54]. According to Stapleton *et al.* [55], organisms responsible for the degradation of PAHs exist in an environment that is extremely acidic (pH 2.0). In a study conducted to determine the effects of seasons and water parameters on PAHs, Hussain *et al*. [56] also observed positive correlation between 3- and 6-ringed PAHs and pH while observing negative correlation between these PAHs and EC as well as DO. According to Boyd *et al.* [57], naphthalene, phenanthrene and fluoranthene were found to increasingly correlate with water DO saturation above 70% (*r* ^2^ > 0.99) over time.

### 5.3. Variation in polycyclic aromatic hydrocarbon molecular weights and their diagnostic ratios

The number of aromatic rings is directly proportional to the molecular weight and toxicity levels of PAHs. It was noted that at four points sampled (Narok-Mau, Narok-Town, Narok-Ewaso Ng’iro and Bomet-Soimet) LMW PAHs dominated, while Narok-Polung’aa, Bomet-Mulot, Bomet-Town and Bomet-Chebole had HMW PAHs (4 and 5 rings). The LMW-PAH : HMW-PAH ratio is a critical toxicity determinant for water bodies [58]. LMW-PAHs have more acute toxicity to aquatic organisms but are not carcinogenic as is the case with HMW-PAHs [58]. In areas with low numbers of PAHs detected, at least half of these were LMW-PAHs. This was attributed to the ubiquitous nature of some LMW-PAHs such as naphthalene, phenanthrene and anthracene—which were present in almost all the samples analysed owing to their enhanced solubility in water compared with HMW-PAHs [59]. Different seasons also affect the variation in these PAHs, as was illustrated by Ambade *et al*. [60]. Out of the four points with these LMW-PAHs, three were in Narok County.

LMW-PAHs result from combustion of biomass materials such as burning of charcoal and agricultural residues before planting, and are termed pyrogenic-based PAHs. On the other hand, HMW-PAHs result from combustion of fossil fuels such as vehicular emissions, and are termed petrogenic-based PAHs. The findings indicate PAH contamination in Narok to mainly arise from pyrogenic sources, as confirmed in table 1. These LMW-PAHs are more water-soluble and thus potentially toxic to aquatic microorganisms [61]. HMW-PAHs such as benzo[*a*]pyrene, benzo[*g,h,i*]perylene, benzo[*a,h*]anthracene and fluoranthene were not common in half of the regions owing to their low solubilities in water. These PAHs were more prevalent in Bomet County—possibly owing to vehicular emissions (petrogenic source). Mobegi & Nyambaka [62] also illustrated the presence of more HMW-PAHs in Ngong River, Nairobi County compared with the LMW-PAHs. In their study, vehicular emissions and industrial discharge of gaseous pollutants were attributed as being the key PAH source leading to the HMW-PAHs. Different anthropological activities can influence these two types of PAHs, as illustrated by Ambade *et al*. [63] during the Covid−19 lockdown. Figure 3 illustrates the distribution of LMW- and HMW-PAHs in the study regions.

The regions with more LMW-PAHs were also found to have *y*_1_ > 0.1, thus indicating that the PAHs at these points are a result of pyrogenic sources (table 1). The most probable pyrogenic source of PAHs in Narok-Mau and ’Narok D’ is charcoal-burning. This activity is extensively practised, especially in Mau Forest (Narok A) owing to the availability of trees and fuel demand in the region. At sampling point Narok B, there is a lot of domestic combustion in households and burning of agricultural residues, leading to PAH emissions. The pyrogenic sources at Bomet-Soimet could also be attributed to emissions arising from combustion of wood fuels in tea factories [64]. Water samples from Narok-Polung’aa, Bomet-Mulot, Bomet-Town and Bomet-Chebole all contained HMW-PAHs in appreciable quantities, arising from petrogenic sources, as was confirmed in table 1. These PAHs mainly arise from petrogenic sources such as vehicular emissions due to traffic [65] in Narok-Polungaa and Bomet-Mulot regions. Benzo[*a*]pyrene, fluoranthene, benzo[*g,h,i*]perylene and benzo[*a,h*]anthracene in the surface waters were attributed to vehicular emissions. Regions with both types of PAH emitters in their proximity, such as Bomet-Mulot, were found to have numerous PAHs in appreciable quantities, as seen in table 2.

### 5.4. Eco-toxicological analysis of the detected polycyclic aromatic hydrocarbons

The PAH levels were analysed using different distribution models to assess the extent of their toxicity (table 3). Both BMC and Q-SAR models exhibited a common trend in the PNEC values of the PAHs in the surface waters. From the BMC values, benzo[*a*]pyrene, anthracene, benzo[*g*,*h*,*i*]perylene and fluoranthene (PNEC < 10) were all found to be toxic at minute concentrations. Similar observations were found for the Q-SAR quantitative structural activity relationship method—which was actually found to be more sensitive to aquatic species compared with the BMC model. Based on this model, benzo[*g,h,i*]perylene, dibenzo[*a,h*]anthracene and benzo[*a*]pyrene (PNEC < 1.0) were found to be the most hazardous PAHs. Naphthalene, phenanthrene and anthracene were found to have more tolerable concentrations based on the two models. The BMC, Q-SAR and classical PNEC trends of the PAHs were found to closely align with the molecular weight of these PAHs. The classical PNEC values for these PAHs revealed ecotoxicity of the PAHs in the order dibenzo[*a,h*]anthracene > benzo[*a*]pyrene > benzo[*g,h,i*]perylene > fluoranthene > anthracene > phenanthrene > naphthalene. These findings are in agreement with those conducted in the Elburgon River, on the northern side of the Mau Forest, as observed by [42]. HMW-PAHs have long persistence in water bodies owing to their immobility, low volatility and higher retention in aquatic organisms, and were found to have higher toxicity levels (benzo[*a*]pyrene and dibenzo[*a,h*]anthracene had the lowest PNEC values of 0.00017 and 0.00014 µg l^−1^, respectively). These PAHs are known to bio-accumulate in aquatic organisms via several exposure routes, including ingestion and dermal [66,67]. LMW-PAHs such as naphthalene can also find their way into humans via inhalation owing to their high volatilities ([68].

The HC_5_ levels of the PAHs detected were in the order: naphthalene > phenanthrene > anthracene > dibenzo[*a,h*]anthracene > fluoranthene > benzo[*g,h,i*]perylene > benzo[*a*]pyrene. Naphthalene, phenanthrene and anthracene are all LMW-PAHs—thus the high HC_5_ levels. The high HC_5_ levels of these PAHs are attributed to, among other factors, their enhanced solubility, which allows them to be taken up by aquatic organisms more easily [53,69]. However, HC_5_ data are obtained from the lethality and immobilization of PAHs in aquatic organisms, and thus do not underlie the chronic effects of the other PAHs [70]—some of which, such as benzo[*g,h,i*]perylene, benzo[*a*]pyrene and dibenzo[*a,h*]anthracene, have more longer-term effects. The hazard of naphthalene toxicity in the water was quite low, with HQ levels of 0.000 for both counties, which is relatively safe. Phenanthrene was absent in both counties (RQ values of 1.288 in Narok and 1.013 in Bomet) and thus posed no risks to aquatic organisms (HQ levels of 0.000). Similar trends were observed for anthracene. Despite being present at low levels, benzo[*a*]pyrene was found to pose a high hazard of 21 and 20 in each county. This is an alarming finding based on the carcinogenic nature of benzo[*a*]pyrene [71]. Other carcinogenic PAHs found to pose danger to water consumption from the two counties were benzo[*g,h,i*]perylene (HQ levels of 4.832 and 8.381) and dibenzo[*a,h*]anthracene (HQ levels of 27.785 and 129.302) in Narok and Bomet counties.

The log-logistic data for dibenzo[*a,h*]anthracene were too large to fit into the plots in figure 2 and were thus plotted separately. This is due to the significantly higher (*p* ≤ 0.05) pollution (RQ) and hazard levels (HQ) of this PAH compared with the rest of the detected PAHs (table 2). The *R* ^2^ values of the species sensitivity distribution (SSD) curves (figure 4) were all above 0.8, except for benzo[*a*]pyrene (*R* ^2^ = 0.7681). This indicates that, apart from these two PAHs, all the rest had a good fit for the PAH toxicity data. The variation in *R* ^2^ can be attributed to varied reference databases used, as well as significantly different concentrations and pollution levels of the PAHs in different rivers (*p* ≤ 0.05*)*. The plots indicate that all the other PAHs posed ecological toxicities at HC_5_ [72] in the surface waters of Narok and Bomet. Benzo[*a*]pyrene was detected in several regions but has a very low HC_5_ value since it is very toxic even at minute concentrations—thus the low correlation coefficient. Naphthalene, fluoranthene and phenanthrene had strong correlation coefficients since they not only were ubiquitous in the surface waters but also had higher HC_5_ reference values compared with the rest of the PAHs.

The two counties would be generally classified as being under potential threat of PAH intoxication based on the toxicity indices provided. Bomet County was more affected compared with Narok County, principally owing to the high number of HMW-PAHs and the petrogenic sources thereof. The region was also prone to some of the identified US EPA PAHs such as benzo[*g,h,i*]perylene and dibenzo[*a,h*]anthracene, thus exposing its water consumers to cancer and other ecotoxicities.

## 6. Conclusions

Seven US EPA priority PAHs were detected at quantifiable levels in the surface waters of Narok and Bomet counties. The concentrations of these PAHs varied, with the highest value being 31.42 µg l^−1^ for dibenzo[*a,h*]anthracene. Benzo[*g,h,i*]perylene, which is a potential carcinogen, was also detected in both counties at above the European Union (EU) permissible levels (10 µg l^−1 and 0.1 µg l−1 respectively)^. There was a positive linear correlation between pH and the concentration levels of PAHs, while EC and DO gave negative linear correlations with PAHs. The majority of the PAHs from Narok County were from pyrogenic sources—mostly combustion of charcoal and other biomass, while those from Bomet County were petrogenic and were attributed to vehicular emissions. Dibenzo[*a,h*]anthracene not only had significantly polluted the surface waters (*p* ≤ 0.05) but also had strong HQs of 128.303 owing to their toxicity, thus posing potential ecological risks. The study therefore recommends urgent water treatment measures prior to use, and the concomitant control of charcoal-burning, stone-mining activities and vehicular emissions near rivers in Narok and Bomet counties. More research should also be conducted on the spatial and temporal variations in PAH levels in aquatic organisms in the two counties.

## Data Availability

Data on the calibration curves and limits of detection of the analyte standards have been uploaded to the Dryad repository [29].
